# A Systematic Review and Meta-Analysis of Unilateral versus Bilateral Pedicle Screw Fixation in Transforaminal Lumbar Interbody Fusion

**DOI:** 10.1371/journal.pone.0087501

**Published:** 2014-01-29

**Authors:** Xu-Qi Hu, Xin-Lei Wu, Cong Xu, Xu-Hao Zheng, Yong-Long Jin, Li-Jun Wu, Xiang-Yang Wang, Hua-Zi Xu, Nai-Feng Tian

**Affiliations:** 1 Department of Orthopaedic Surgery, Second Affiliated Hospital of Wenzhou Medical University, Wenzhou, Zhejiang, China; 2 Institute of Digitized Medicine, Wenzhou Medical University, Wenzhou, Zhejiang, China; Georgia Regents University, United States of America

## Abstract

**Background:**

Transforaminal lumbar interbody fusion (TLIF) has become one of the most widely used procedures for lumbar spinal disorders. However, it is still unclear whether TLIF with unilateral pedicle screw (PS) fixation is as effective as that with bilateral PS fixation. We performed a meta-analysis of the literatures and aimed to gain a better understanding of whether TLIF with unilateral PS fixation was safe and effective for lumbar diseases.

**Methodology/Principal Findings:**

We systematically searched Ovid, Springer, and Medline databases for relevant randomized controlled trials (RCTs) that compared the clinical and radiological outcomes of unilateral versus bilateral PS fixation in TLIF. Risk of bias in included studies was assessed using the Cochrane Risk of Bias tool. We generated pooled risk ratios or weighted mean differences across studies. According to our predefined inclusion criteria, seven RCTs with a total of 441 patients were included in this study. Baseline characteristics were similar between the unilateral and bilateral groups. Our meta-analysis showed that no significant difference was detected between the two groups in terms of postoperative clinical function, fusion status, reoperation rate, complication rate, and hospital stay (p>0.05). Pooled estimates revealed that the unilateral group was associated with significantly reduced implant cost, operative time and blood loss (p<0.05).

**Conclusions/Significances:**

Our meta-analysis suggested TLIF with unilateral PS fixation was as safe and effective as that with bilateral PS fixation for lumbar diseases in selected patients. Despite these findings, our meta-analysis was based on studies with small sample size and different study characteristics that might lead to the inconsistent results such as various functional outcomes among the included studies. Therefore, high-quality randomized controlled trials with larger sample size are also needed to further clarify these issues and to provide the long-term outcomes.

## Introduction

Since Harms et al. [1] firstly introduced the technique in 1982, transforaminal lumbar interbody fusion (TLIF) has become a popular procedure for various lumbar disorders. The TLIF procedure reduces the retraction of the dural sac and nerve roots, thus decreasing the risk of potential complications like dural tears and neurological injury [2], [3]. Moreover, TLIF preserves the interlaminar surface of the contralateral side that can be used as additional fusion site [2], [4].

Traditionally, standard TLIF is performed with bilateral PS fixation. It provided rigid fixation and excellent clinical outcomes [3]–[7]. Recently, TLIF with unilateral PS fixation has been developed, which further reduces the blood loss, surgical time and tissue trauma [8]–[11]. Biomechanical studies showed that unilateral PS fixation was potentially less stable than bilateral PS fixation [12]. Finite element analysis also demonstrated similar results and the authors recommended the supplemental use of a contralateral facet screw [13]. Though less rigid biomechanically, unilateral fixation in TLIF may be sufficient for achieving radiographic fusion and satisfactory clinical outcomes. Many surgeons reported that TLIF with unilateral PS fixation obtained favorable clinical results and recommended it as an option for appropriately selected patients [9], [10], [14], [15].

Currently, an increasing number of studies have been conducted to compare the clinical and radiological outcomes of unilateral versus bilateral PS fixation in TLIF. By summarizing the evidence from radomized controlled trials (RCTs), we performed this meta-analysis and aimed to gain a better understanding of whether TLIF with unilateral PS fixation was as safe and effective as that with bilateral PS fixation.

## Methods

### Search strategy and inclusion criteria

This meta-analysis was performed in accordance with the preferred reporting items for systematic reviews and meta-analyses (PRISMA) guidelines (Checklist S1). A systematic literature search was conducted up to August 2013 using Ovid, Springer, and Medline databases. We screened the title and abstract by combining the term “unilateral” with each of the following keywords: “transforaminal lumbar interbody fusion”, “posterior lumbar interbody fusion”, “TLIF”, and “PLIF”. Articles were limited to those published in the English. Additionally, a comprehensive search of reference lists of selected articles and relevant reviews was also performed. Unpublished data were not reviewed. The following eligibility criteria were used in selecting articles: (1) Randomized controlled trial. (2) The study compared the clinical and/or radiological outcomes of TLIF with unilateral versus bilateral PS fixation. (3) The study population consisted of adult patients suffering from degenerative lumbar disease. (4) Peer reviewed full text. Articles were excluded if they had any of following characteristics: (1) Patients with spinal deformities, traumas, or spinal tumors. (2) Patients suffered from systematic disorders such as active infection, metabolic disease, severe osteoporosis, and symptomatic vascular disease. (3) Repeated studies.

### Data extraction

Data was independently extracted by two reviewers based on the following categories: (1) Basic characteristics such as year of publication, age, gender, enrolled number, follow-up duration, and follow-up rate. (2) Surgical information, including surgical segment and levels, instrumentation, and graft type. (3) Primary outcomes, consisting of postoperative functional outcome, nonunion, complication, and reoperation. (4) Secondary outcomes such as operative time, blood loss, hospital stay, and implant cost. Disagreement between the reviewers was resolved by consensus with a third reviewer.

### Risk of Bias Assessment

To assess the risk of bias of the included studies, the Cochrane Handbook for Systematic Reviews of Interventions was applied, including: (1) Random sequence generation. (2) Allocation concealment. (3) Blinding of participants and personnel. (4) Blinding of outcome assessment. (5) Incomplete outcome data. (6) Selective reporting. (7) Other bias. Reviewers' judgments were categorized as low risk of bias, high risk of bias, or unclear risk of bias.

### Statistical Analysis

Meta-analysis was performed using Review Manager 5.0 software (Cochrane Collaboration, Oxford, UK). Weighted mean differences (WMD) were calculated for continuous outcomes and risk ratios (RR) for binary outcomes, along with 95% confidence intervals (CIs). The level of significance was set at p<0.05. Heterogeneity was evaluated using the χ^2^ test and I^2^ statistics. (Heterogeneity was detected when p<0.10 or I^2^>50%). Fixed-effect models were applied unless statistical heterogeneity was significant, in which case random-effect models were used. Data in non-standard forms were converted according to the method described by Cochrane handbook for systematic reviews of interventions. We utilized funnel plots to assess the possibility of publication bias. The sensitivity analysis was performed to test the strength and robustness of pooled results by sequential omission of individual studies.

## Results

### Literature Search

Our search strategy (Figure S1) initially yielded 183 citations (n = 90 by Medline, n = 27 by Ovid, and n = 66 by Springer), of which 149 were screened after removal of duplicated records (n = 34). Review of titles and abstracts resulted in exclusion of 107 studies that covered inappropriate topics. The full text of remaining 42 papers were obtained and assessed for eligibility. 35 of them were further removed according to predefined inclusion/exclusion criteria. Finally, seven RCTs were selected and analyzed [16]–[22].

### Risk of Bias Assessment

The risk of bias assessment of all included studies [16]–[22] was described in Table 1. Five [16]–[19], [22] studies described adequate methods of random sequence generation. Allocation concealment was well described in only two trials [16], [22]. Due to the nature of the trials, it was impossible to perform blinding of participants and personnel. Four studies [18]–[20], [22] reported blinding of outcome assessment while the other three [16], [17], [21] did not. A total of nine patients were lost to follow-up [17], [20], [21]. Since the lost data was small in each study, we regarded these studies as low risk of incomplete outcome data addressed. We also consider all of the included studies as low risk of selective reporting for they provided the outcomes in detail. There was no other bias like funding bias or baseline imbalance in these studies.

**Table 1:** Risk of bias assessment of the included studies.

Risk of bias assessment	Feng 2011	Aoki 2012	Xie 2012	Xue 2012	Dahdaleh 2013	Choi 2013	Zhang 2013
Random sequence generation	Low	Low	Low	Low	Unclear	Unclear	Low
Allocation concealment	Low	Unclear	Unclear	Unclear	Unclear	Unclear	Low
Blinding of participants and personnel	High	High	High	High	High	High	High
Blinding of outcome assessment	Unclear	Unclear	Low	Low	Unclear	Low	Low
Incomplete outcome data addressed	Low	Low	Low	Low	Low	Low	Low
Selective reporting	Low	Low	Low	Low	Low	Low	Low
Free of other bias	Low	Low	Low	Low	Low	Low	Low

### Study Characteristics

The basic information of the seven included studies was presented in Table 2. Statistically similar baseline was observed between the unilateral and bilateral groups (Table 3). A total of 442 patients were evaluated (mean age of 58.5 years). The fusion segment was located at L3-S1. Comparison of preoperative diagnosis was performed in four papers, with no significant difference [16], [18], [19], [22]. Except for one trial [21], there was no significant difference in preoperative clinical function between the two groups. Three studies [18], [19], [22] reported lumbar fusion in two spinal levels. Minimally invasive transforaminal lumbar interbody fusion (MIS-TLIF) was used in two trials [20], [21]. Cages supplemented with rhBMP were applied in Dahdaleh's study [21]. Aoki reported that 2 cages were implanted in the bilateral group [17]. One cage per level was used in the left studies.

**Table 2:** Characteristics of the included trials.

Characteristic	Feng 2011	Aoki 2012	Xie 2012	Xue 2012	Dahdaleh 2013	Choi 2013	Zhang 2013
Basic information							
Year of publication	2011	2012	2012	2009	2013	2013	2013
Study design	RCT	RCT	RCT	RCT	RCT	RCT	RCT
No. enrolled Patients (Uni vs Bi)	20∶20	25∶25	56∶52	37∶43	20∶21	26∶28	33∶35
Diagnosis	LSS, LS grade I, II	LS grade I, II	LSS, RLDH, SDDD	LSS, LS, LDH, RLDH, DLBP	LS grade I, II	LSS, LS, LDH, RLDH,	LSS, LS, SDDD, FBS
No. followed patients (Uni vs Bi)	20∶20	24∶23	56∶52	37∶43	16∶20	26∶27	33∶35
Follow-up rate (%; Uni vs Bi)	100∶100	96∶92	100∶100	100∶100	80∶95.2	100∶96.4	100∶100
Mean follow-up time (mo; Uni vs Bi)	3∶3	31.0∶31.2	>36	25.3	11.4∶12.4	27.5∶28.9	25.6
Mean age (yr; Uni vs Bi)	53.8∶53.2	66.2∶65.6	56.2∶55.0	57.1∶58.2	62.2∶57.3	53.6∶56.2	59.4∶55.7
Gender (% male; Uni vs Bi)	40∶50	32∶48	42.9∶46.2	45.9∶41.9	25∶30	46.2∶33.3	57.6∶71.4
Surgical information							
MIS-TLIF	No	No	No	No	Yes	Yes	No
Spinal segment	L3-S1	L3-S1	L3-S1	L3-S1	L3-S1	L3-S1	L3-S1
No. fused levels	1 level	1 level	1/2 levels	1/2 levels	1 level	1 level	2 levels
Graft use	1 cage	1 cage in uni; 2 cages in bi	1 cage	1 cage	1 cage + rhBMP	1 cage	1 cage

RCT: randomized controlled trial. Uni vs Bi: the unilateral fixation group vs the bilateral fixation group. MIS-TLIF: minimally invasive surgery for transforaminal lumbar interbody fusion. In this study, MIS-TLIF refers to those assisted by a tubular retractor system. LSS: lumbar spinal stenosis. LS: lumbar spondylolisthesis. LDH: lumbar disc herniation. RLDH: recurrent lumbar disc herniation. SDDD: symptomatic degenerative disc disease. DLBP: discogenic low back pain. FBS: failed back surgery.

**Table 3:** Comparison of baseline characteristics between the unilateral fixation group and bilateral fixation group.

Characteristic	Feng 2011	Aoki 2012	Xie 2012	Xue 2012	Dahdaleh 2013	Choi 2013	Zhang 2013
Gender	*	*	*	*	*	*	*
Mean age	*	*	*	*	*	*	*
Follow-up time	*	*	NA	NA	*	*	NA
Fusion segment	*	*	*	*	*	*	*
No. fused levels	*	*	*	*	*	*	*
Preoperative diagnosis	*	NA	*	*	NA	NA	*
Preoperative pain score (VAS)	*	*	*	*	*	*	*
Preoperative functional score (JOA ODI)	*	*	*	*	*	*	*

NA: not available. VAS: visual analog scale. JOA: Japanese Orthopedic Association. ODI: Oswestry Disability Index.

* Statistically insignificant (p>.0.05).

### Postoperative clinical function

**Figure 1:**
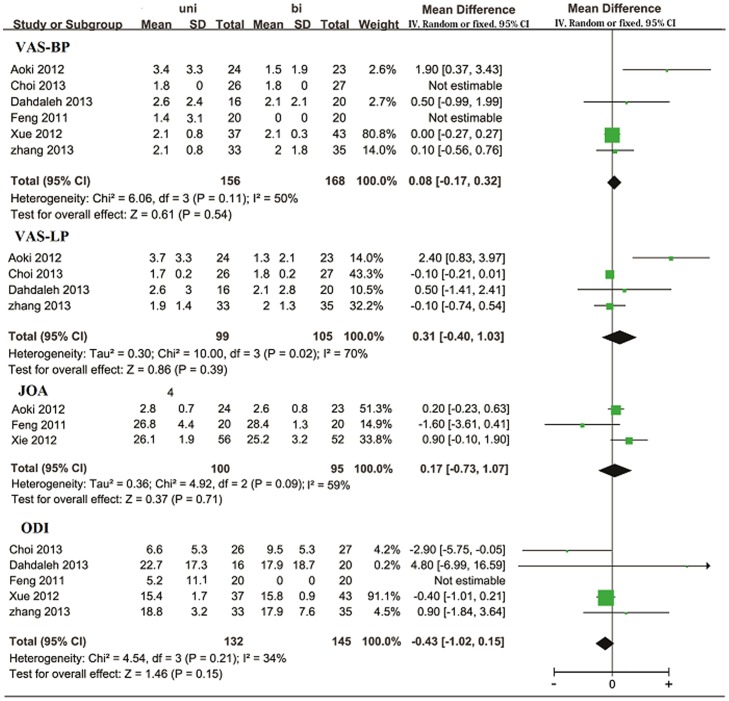
Forest plot illustrating postoperative VAS, JOA, and ODI score of meta-analysis comparing unilateral with bilateral PS fixation in TLIF.

The most frequently used methods to assess the clinical function were visual analog scale (VAS), Japanese Orthopedic Association (JOA) scores, and Oswestry Disability Index (ODI). VAS for back pain was available in six trials [16], [17], [19]–[22]. Meta-analysis did not reveal any significant difference (WMD  = 0.08, 95% CI: −0.17–0.32, p = 0.54; I^2^ = 50%, p = 0.11) (Figure 1). VAS for leg pain was available in four trials [17], [20]–[22]. The pooled estimate also show no significant difference between the two arms (WMD  = 0.31, 95% CI: −0.40–1.03, p = 0.39; I^2^ = 70%, p = 0.02) (Figure 1). JOA scores were reported in three trials [16]–[18]. Overall, no significant intergroup difference was detected (WMD  = 0.17, 95% CI: −0.37–1.07, p = 0.71; I^2^ = 59%, p = 0.09) (Figure 1). ODI scores were available in five trials [16], [19]–[22]. The pooled data did not reveal any significant difference between the two groups (WMD  = −0.43, 95% CI: −1.02–0.15, p = 0.15; I^2^ = 34%, p = 0.21) (Figure 1). SF-36 scores were applied in three trials with no significant intergroup difference [18], [21], [22]. Aoki et al. [17] assessed postoperative clinical function by JOABPEQ scores. Xue et al. [19] used mProlo scores to evaluate the clinical function. Neither of them observed any significant difference between the two groups.

### Nonunion

**Figure 2:**
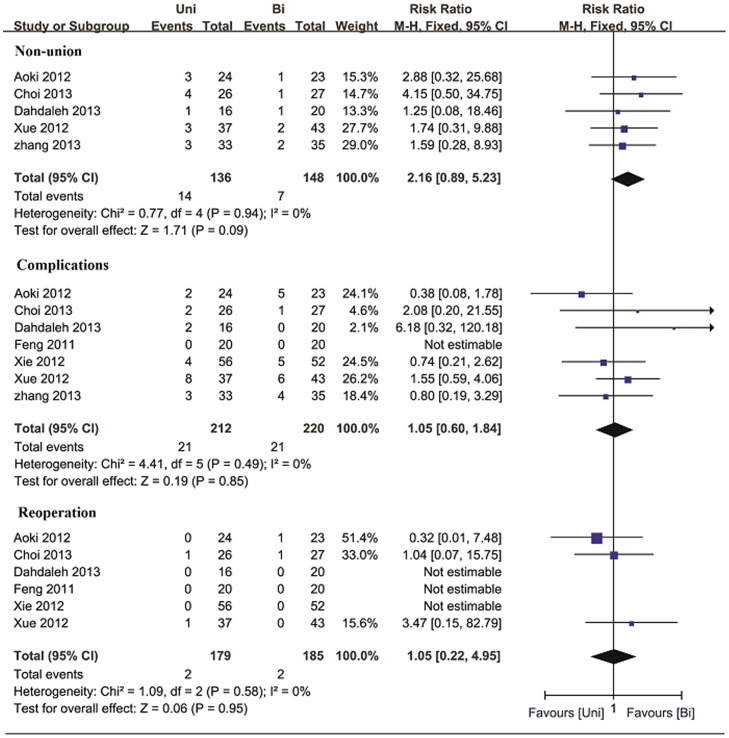
Forest plot illustrating non-union rate, complication rate, and reoperation rate of meta-analysis comparing unilateral with bilateral PS fixation in TLIF.

The nonunion rate was assessed in five studies [17], [19]–[22], with no significant difference between the unilateral and bilateral groups. The pooled estimate also demonstrated no significant intergroup difference. (RR  = 2.16, 95% CI: 0.89–5.23, p = 0.09; I^2^ = 0%, p = 0.94) (Figure 2).

### Complication and reoperation

Data regarding complications were provided in all included studies. There was no evidence of significant heterogeneity (I^2^ = 0%, p = 0.49). The pooled complication rate also demonstrated no evidence of significant difference between the two groups (RR  = 1.05, 95% CI: 0.60–1.84, p = 0.85) (Figure 2). Data of reoperation was available in six studies. Meta-analysis of reoperation rate revealed that the difference was statistically insignificant (RR  = 1.05, 95% CI: 0.22–4.95, p = 0.95; I^2^ = 0%, p = 0.58) (Figure 2).

### Operative time

**Figure 3:**
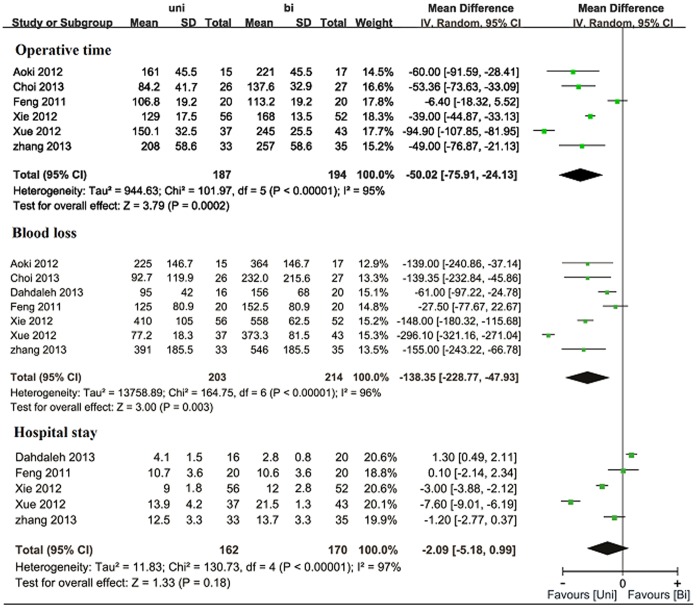
Forest plot illustrating operative time, blood loss, and hospital stay of meta-analysis comparing unilateral with bilateral PS fixation in TLIF.

Six trials reported the operative time [16]–[20], [22]. Five trials [17]–[20], [22] showed the operative time was significant longer in the bilateral group than that in the unilateral group. The pooled estimate demonstrated significant difference between the two groups (WMD  = −50.02, 95% CI: −75.91–−24.13, p<0.001). Significant heterogeneity was detected (I^2^ = 95%; p<0.001) (Figure 3).

### Blood loss

All the included trials assessed blood loss. Six trials [17]–[22] reported significantly reduced blood loss in the unilateral group. Pooled analysis revealed that blood loss was significantly less in the unilateral group (WMD = −138.35, 95% CI: −228.77 –−47.93, p = 0.003; I^2^ = 96%; p<0.001) (Figure 3).

### Hospital stay and implant cost

Five trials reported data of hospital stay [16], [18], [19], [21], [22]. Statistical heterogeneity was detected (I^2^ = 97%; p<0.001). Pooling of relevant data revealed statistically insignificant difference (WMD  = −2.09, 95% CI: −5.18–0.99, p = 0.18) (Figure 3). Three studies reported significantly higher implant cost in the bilateral group [16], [19], [22]. The pooled estimate was statistically significant in favor of the unilateral group (p<0.001).

### Publication bias and sensitivity analysis

**Figure 4:**
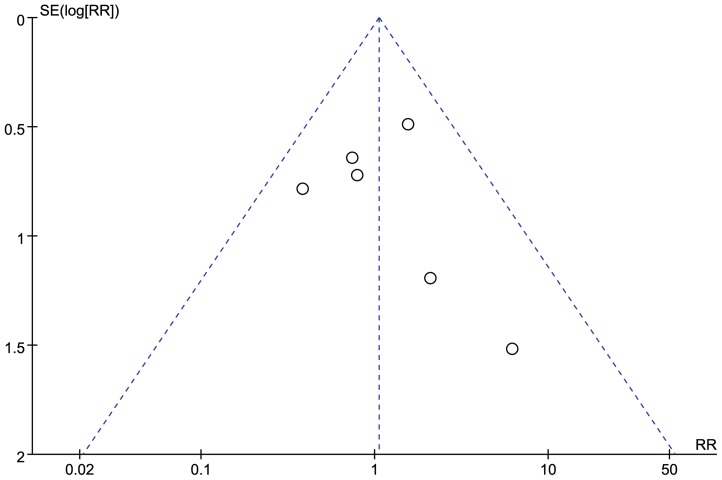
Funnel plot of total complication rate.

A funnel plot of the studies that reported the incidence of complications is shown in Figure 4. All studies lied within the 95% CI and were distributed evenly about the vertical, implying minimal publication bias. Sensitivity analysis was conducted by reanalyzing our data after sequential omission of individual studies. Pooled results did not yield any significant difference by omitting any single study data.

## Discussion

Spinal fusion with pedicle screws is widely used [23]. However, the choice between unilateral or bilateral PS fixation in TLIF is still controversial. Our meta-analysis suggested that no significant difference was detected between the unilateral PS fixation group and the bilateral PS fixation group in terms of postoperative clinical function, fusion status, reoperation rate, complication rate, and hospital stay. The clinical significance is that TLIF with unilateral PS fixation may be suitable for appropriately selected patients.

Some biomechanical studies revealed that unilateral PS fixation was less stable than bilateral PS fixation in TLIF, especially in resisting axial rotation and lateral bending. [12], [24], [25]. Less biomechanical stability in unilateral instrumentation might have an impact on the fusion rate. We found the included trials consistently showed that the non-union rate in the unilateral group was slightly higher than that in the bilateral group. After pooling of individual data, the total non-union rate was 10.29% in unilateral group and was 4.73% in bilateral group. Although the pooled estimate was statistically insignificant, the significant p value ( = 0.09) increased, which meant the result might be altered if the study number or study sample size increased. During our review, we observed that two trials had demonstrated a significantly increased incidence of postoperative scoliosis in the unilateral group [20], [22] which could be explained by the difference in biomechanical properties. Nevertheless, Choi found that the patients with postoperative scoliosis had a similar fusion rate and clinical result as the patients without scoliosis [20]. Furthermore, the radiological outcomes were mostly obtained from short term follow-ups. Thus, further large randomized controlled trials with long term follow-up are still needed to confirm these results.

Four patients received reoperation in the included trials, with no significant difference between the two groups. One of the main reasons for reoperation was cage migration. A previous retrospective study reported that the use of a bullet-shaped cage, undersized cage, higher PDH, and the presence of scoliotic curvature were possible risk factors for cage migration [26]. Here, cage migration was reported in two trials, in which bullet-shaped cages were used [17], [20]. Moreover, MIS-TLIF technique with a tubular retractor may impose restriction on the cage size and location, potentially increasing the incidence of cage migration [20].

The adjacent segment disease (ASD) has been demonstrated as a common complication in lumbar fusion surgeries [27], [28]. Theoretically, the unilateral fixation was less stiff, which might prevent the adjacent segment from early degeneration. Toyone et al. [29] observed a lower incidence of adjacent segment degeneration in PLIF with unilateral PS fixation than that in PLIF with bilateral PS fixation during a 5 years of follow-up. In current review, only Choi et al. [20] reported a case of upper segment disc herniation in the bilateral group. The possible reason of low prevalence of ASD in both groups might be that the follow-up time in these trials was not long enough. Therefore, long-term follow-up is essential for clarifying whether there is a difference on prevalence of ASD between the two treated methods [23].

We observed that both the unilateral and the bilateral groups achieved significantly improved functional outcomes such as VAS, JOA, ODI, and so on. Thus, both modalities were efficient. Considering that pooled estimates did not reveal significantly difference between the two groups, other surgical outcomes should be taken into account when we decide to apply which method. Our study showed that the operative time, blood loss, and implant cost was significantly less in the unilateral group. This was because the unilateral PS fixation avoided surgical exposure of the contralateral side and employed a much less invasive approach. Therefore, it facilitated the early recovery and rehabilitation of the patients [22].

There are a number of limitations in this study. First, only seven small trials were included in our study. Thus, the analysis was based on only 441 patients. Second, some baseline characteristics were different among the trials, such as segment and levels of fusion, cage use, surgical technique, and follow-up duration. Aoki et al [17] used one cage in the unilateral group, but implanted two cages in the bilateral group. Dahdaleh et al applied rhBMP as the bone fusion enhancer [21]. In some studies [18], [19], [22], 2-level TLIF was performed, while the other studies involved only single level fusion. These may have potential affects on surgical outcomes. Third, we found the definition of a complication was of great difference. There might be bias if we pooled this estimate according to the definition in each study. The main reported complications included pedicle screw loosening or malposition, cage migration, nonunion, neurological injury, dural tear, infection, and deep venous thrombosis. Lastly, short-term clinical and radiological outcomes may limit the application of the procedure. Anyhow, this is the first meta-analysis of RCTs that has compared the clinical and radiological outcomes between unilaterally and bilaterally instrumented TLIF. Pooled analysis shows TLIF with unilateral PS fixation is a safe and effective method to treat lumbar disease in selected patients. However, high quality RCTs with large sample size and long-term follow-up are still needed to further confirm this conclusion.

## Supporting Information

Figure S1 **Selection of relevant publications, reasons for exclusion.**
(TIF)

Checklist S1 **PRISMA Checklist.**
(DOC)
