# Supplementary figures and images for: A Systematic Review and Meta-Analysis of Unilateral versus Bilateral Pedicle Screw Fixation in Transforaminal Lumbar Interbody Fusion
Source: PLoS One. 2014 Jan 29;9(1):e87501. doi: 10.1371/journal.pone.0087501 (PMC3906181; doi:10.1371/journal.pone.0087501)

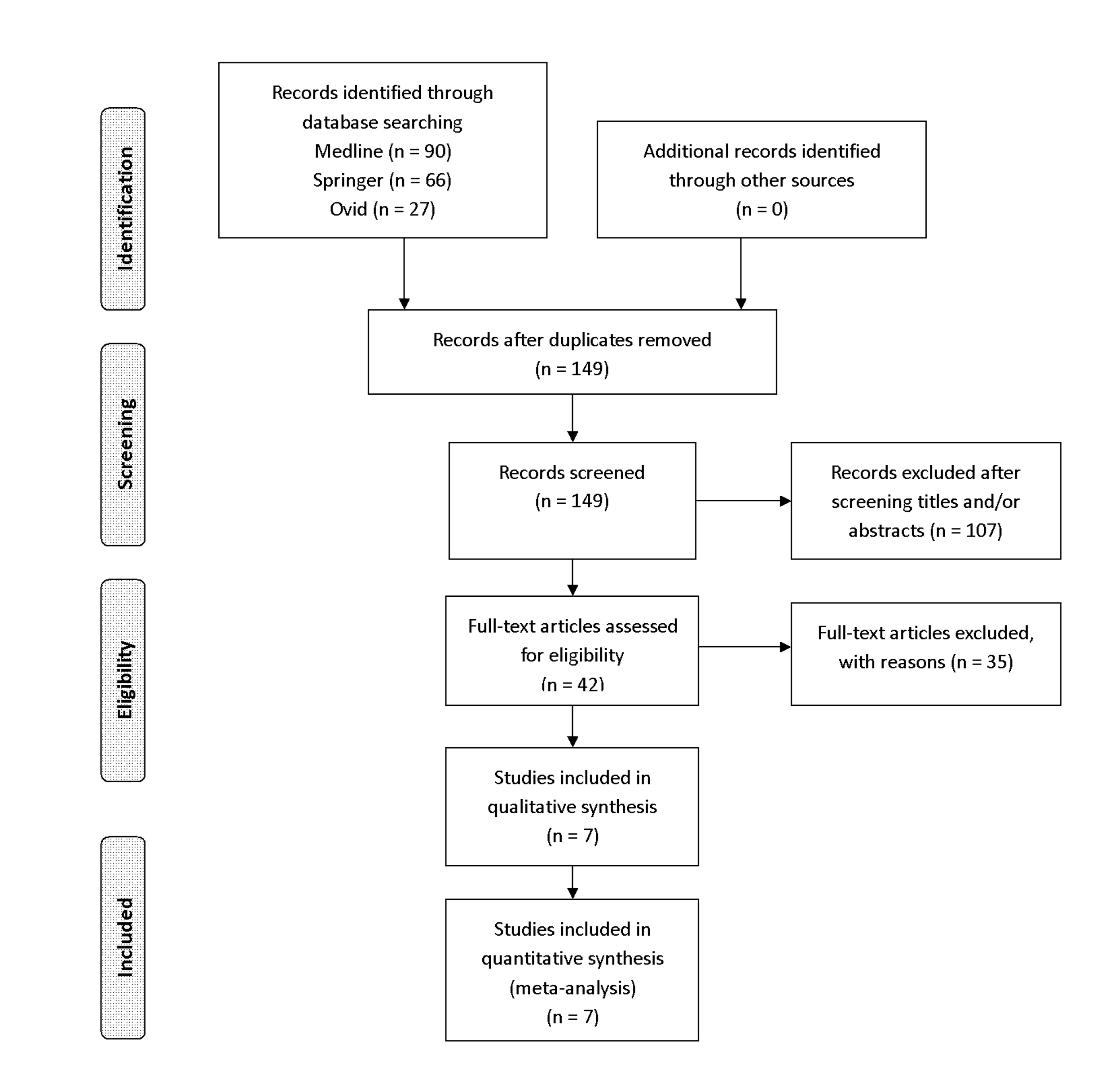

Supplement: Figure S1 — Selection of relevant publications, reasons for exclusion. (TIF) [file pone.0087501.s001.tif]
